# Supplementary material for: A high-resolution mRNA expression time course of embryonic development in zebrafish
Source: eLife. 2017 Nov 16;6:e30860. doi: 10.7554/eLife.30860 (PMC5690287; doi:10.7554/eLife.30860)
Supplement: Supplementary file 6. [file elife-30860-supp6.zip › biolayout-clusters-files/Cluster079-genes.html]

Cluster079


# Cluster079: Genes

| Ensembl ID | Gene Name | Chr | Start | End | Biotype |
| --- | --- | --- | --- | --- | --- |
| ENSDARG00000075024 | ENSDARG00000075024 | 23 | 44681181 | 44705411 | protein\_coding |
| ENSDARG00000059693 | adam19a | 7 | 60992892 | 61221595 | protein\_coding |
| ENSDARG00000063612 | antxr1c | 12 | 2642570 | 2709913 | protein\_coding |
| ENSDARG00000056530 | cpamd8 | 22 | 21221781 | 21279269 | protein\_coding |
| ENSDARG00000057984 | eps8l1 | 3 | 16363419 | 16387641 | protein\_coding |
| ENSDARG00000018441 | heg1 | 9 | 38875076 | 38897715 | protein\_coding |
| ENSDARG00000039452 | hk1 | 13 | 22842438 | 22877363 | protein\_coding |
| ENSDARG00000103370 | lrrc8da | 2 | 22839623 | 22846600 | protein\_coding |
| ENSDARG00000076533 | nfe2l1b | 12 | 28739508 | 28751193 | protein\_coding |
| ENSDARG00000076594 | pcdh12 | 14 | 16003187 | 16022678 | protein\_coding |
| ENSDARG00000075253 | pik3cb | 15 | 7068022 | 7165080 | protein\_coding |
| ENSDARG00000005754 | ptprfb | 2 | 17907221 | 18275975 | protein\_coding |
| ENSDARG00000005593 | rxrga | 2 | 21067741 | 21118869 | processed\_transcript |
| ENSDARG00000032161 | srgap2 | 11 | 21246118 | 21424608 | protein\_coding |
| ENSDARG00000059483 | tead1b | 7 | 65532408 | 65701173 | protein\_coding |
| ENSDARG00000031164 | tuba8l2 | 1 | 4704671 | 4721311 | protein\_coding |
